# Extended criteria donor organ use for heart-lung transplantation in the modern era

**DOI:** 10.1016/j.clinsp.2023.100205

**Published:** 2023-04-28

**Authors:** Noah Weingarten, Amit Iyengar, David Alan Herbst, Mark Helmers, Danika Meldrum, Sara Guevara-Plunkett, Jessica Dominic, Pavan Atluri

**Affiliations:** Division of Cardiovascular Surgery, Department of Surgery, University of Pennsylvania, Philadelphia, USA

**Keywords:** Organ donor management, Heart-lung transplantation, Heart transplantation, Lung transplantation

## Abstract

**Background:**

Demand for donor hearts and lungs exceeds their supply. Extended Criteria Donor (ECD) organs are used to help meet this demand, but their impact on heart-lung transplantation outcomes is poorly characterized.

**Methods and results:**

: The United Network for Organ Sharing was queried for data on adult heart-lung transplantation recipients (*n* = 447) from 2005‒2021. Recipients were stratified based on whether they received ECD hearts and/or lungs. Morbidity was analyzed using Kruskal-Wallis, chi-square, and Fisher's exact tests. Mortality was analyzed using Kaplan-Meier estimation, log-rank tests and Cox regression. Sixty-five (14.5%) patients received two ECD organs, 134 (30.0%) received only an ECD lung, and 65 (14.5%) only an ECD heart. Recipients of two ECD organs were older, more likely to have diabetes, and more likely transplanted from 2015‒2021 (*p* < 0.05). Groups did not differ by pre-transplant diagnosis, intensive care unit disposition, life support use, or hemodynamics. Group five-year survival rates ranged from 54.5% to 63.2% (*p* = 0.428). Groups did not differ by 30-day mortality, strokes, graft rejection, or hospital length of stay.

**Conclusions:**

: Using ECD hearts and/or lungs for heart-lung transplantation is not associated with increased mortality and is a safe strategy for increasing donor organ supply in this complex patient population.

## Highlights

• Extended criteria donor organs in heart-lung transplant do not worsen mortality.
• Advanced donor age and P/F ratio remain predictive of mortality.
• Extended criteria donor organ use is safe in appropriately chosen patients.

## Introduction

Demand for donor hearts and lungs continues to exceed their supply. One strategy to increase supply is the use of Extended Criteria Donor (ECD) organs. While there is no consensus on what the extended criteria for hearts or lungs are, previously published lists have considerable overlap and tend to include increased donor age, ischemic time, and other risk factors for end-organ dysfunction [1,2]. Several transplant centers have found comparable outcomes between recipients of carefully selected ECD hearts and recipients of Standard Criteria Donor (SCD) hearts [1]. The extended heart criteria found to minimally impact morbidity and mortality include age over 50 [1], norepinephrine requirement [3], hepatitis C positivity [1,4], left ventricular ejection fraction < 50% [5], and donor/recipient weight ratio less than 0.80 [1]. Extended lung criteria found to minimally impact these outcomes include age over 50 [6], tobacco use history [7], and hepatitis C positivity [8].

Simultaneous Heart-Lung Transplantation (HLTx) remains the preferred treatment for concomitant end-stage cardiac and pulmonary dysfunction. Yet, finding organs for these very ill patients can be challenging. While the benefit of using carefully selected ECD organs for single-organ heart and lung transplantations has been shown, it is unclear what effect ECD organs have on HLTx outcomes. Furthermore, the complexities of this patient subset, including the presence of Eisenmenger syndrome with an unrepairable cardiac defect, differ from those of single-organ heart or lung transplantation [9]. It remains to be determined whether transplant recipients with these unique pathologies tolerate ECD organs as well as recipients of single-organ thoracic transplantations. Further, no study has assessed which ECD criteria, if any, contribute to mortality in the setting of HLTx. Lastly, it is unknown how often ECD organs are used in the setting of HLTx and whether their use has increased in response to accumulating data on the tolerability of ECD organs in single-organ thoracic transplants.

The authors undertook this study to 1) Assess the effect of ECD organs on morbidity and mortality in the setting of HLTx and 2) Assess the prevalence of ECD heart and lung use in HLTx over time.

## Materials and methods

The United Network for Organ Sharing (UNOS) thoracic database was queried for all patients aged 18 or older who received HLTx from January 1, 2005, through July 19, 2021. 2005 was selected to exclude transplantations prior to the implementation of lung allocation scores. Patients with unknown mortality status at the last follow-up were excluded. Each HLTx recipient was assigned to one of four groups based on donor organ status. The four groups were recipients of either two Standard Criteria Donor (SCD) organs, two Extended Criteria Donor (ECD) organs, an ECD heart and SCD lung, or an ECD lung and SCD heart. Donor hearts and lungs were determined to be ECD organs if they satisfied two ECD criteria (Table 1). ECD criteria were adapted from the literature describing extended criteria in hearts [1,3, 4, 5,10, 11, 12, 13, 14, 15, 16, 17] and lungs [2,6, 7, 8,17].

**Table 1:** List of extended donor criteria for hearts and lungs.

Extended heart criteria	Extended lung criteria
Age ≥ 55 [1,14,16,17]	Age ≥ 55 [6,9,17]
Cold ischemic time > 360 min [1,13,14]	Infiltrates on chest radiograph [2,9,17]
Donor/recipient weight ratio 〈0.80 or〉 ≥ 1.30 [1,13,17,20]	P/F ratio < 300 [2,9,17]
Hepatitis C positivity [1,4,8,16]	Purulent secretions on bronchoscopy [2,9,17]
Drug use history [16]	Cigarette use history (> 20 pack-years) [2,7,9,17]
Renal insufficiency (serum creatinine > 2.0 mg/dL) [11]	
Left ventricular ejection fraction < 50% [5,13,17]	
Donation after circulatory death [12,13]	

P/F ratio, Ratio of arterial oxygen partial pressure to a fraction of inspired oxygen.

Baseline characteristics, morbidity, and mortality data are reported for all donor recipients and compared by donor organ status. Predicted total lung capacity was calculated from height using previously described methods [18]. Categorical variables are expressed as frequency (%) and continuous variables are presented as median [interquartile range]. Comparisons between donor organ status groups were performed using chi-square for categorical variables with group sample sizes greater than 5, Fisher's exact test for categorical variables with sample sizes less than or equal to 5, and Kruskal-Wallis for continuous variables that are non-parametrically distributed. Parametricity was assessed for each continuous variable using the Shapiro-Wilk test. Mortality was censored at five years and analyzed using Kaplan-Meier estimation. Mortality comparisons between donor organ status groups were performed using a log-rank test. A Cox proportional hazards regression model was used to determine predictors of five-year mortality. Variables used in the Cox regression model included each extended donor criterion, as well as each recipient and donor characteristic that differed significantly between donor organ groups on univariate analysis. All statistical analysis was performed using STATA/MP 17.0 software (StataCorp LLC, College Station, TX).

The University of Pennsylvania Institutional Review Board deemed the study to be not human subjects research and waived the need for patients’ written informed consent (protocol #: 850952, approval date: March 10, 2022). The study was completed in conformity with STROBE Statement guidelines.

## Results

**Fig. 1:**
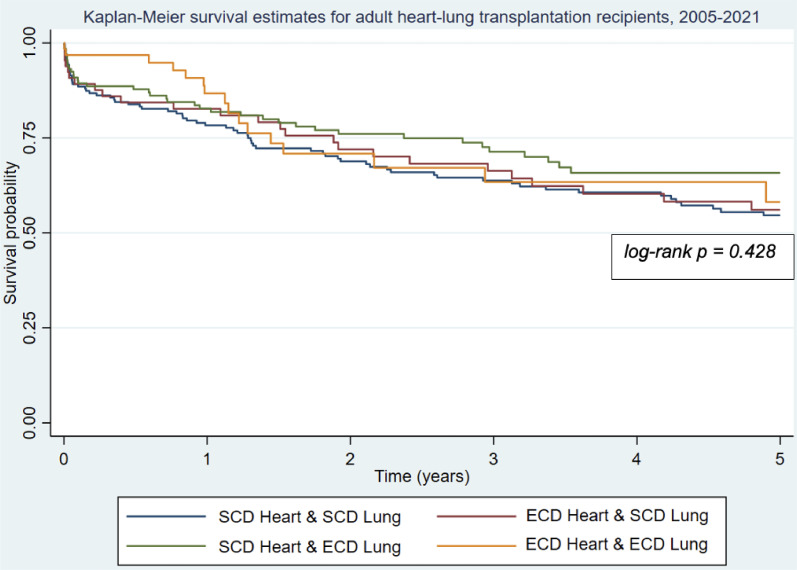
Kaplan-Meier survival estimates for adult heart-lung transplantation recipients from 2005‒2021, stratified by donor organ status. ECD, Extended Criteria Donor; SCD, Standard Criteria Donor.

447 adults undergoing simultaneous HLTx from 2005 to 2021 were analyzed (Fig. 1). 232 (51.9%) were female. The median recipient age was 45. The most common recipient diagnosis was primary pulmonary hypertension (*n* = 111, 24.9%). The largest cohort of recipients (*n* = 178, 39.8%) received two SCD organs. 134 (30.0%) received only an ECD lung, 70 (15.7%) received only an ECD heart, and 65 (14.5%) received both an ECD lung and an ECD heart.

The four donor organ status groups differed by recipient characteristics including age, diabetes, cytomegalovirus positivity, and year of transplant, as well as donor characteristics including sex, age, body mass index, hepatitis C status, drug use, cigarette use history (> 20 pack-years), inotrope need, serum creatinine, infiltrates on chest radiographs, purulent secretions on bronchoscopy, a ratio of arterial oxygen partial pressure to the fraction of inspired oxygen (P/F ratio), and circulatory death (*p* < 0.05) (Table 2, Table 3, Table 4). Recipients of two ECD organs were older, more likely to have diabetes, and more likely transplanted in the recent era (2015‒2021). Recipients of ECD hearts but SCD lungs were more likely than other groups to be transplanted from 2005‒2009. Organ donor status groups did not differ by pre-operative recipient diagnosis, intensive care unit disposition, hemodynamics, waiting list time, ischemic time, or need for life support devices including dialysis, mechanical ventilation, Extracorporeal Membrane Oxygenation (ECMO), ventricular assist devices, and intra-aortic balloon bumps.

**Table 2:** Pre-transplant demographics of adult heart-lung transplantation recipients from 2005‒2021, stratified by donor organ status.

	All patients (*n* = 447)	SCD heart and lung recipients (*n* = 178)	ECD heart only recipients (*n* = 70)	ECD lung only recipients (*n* = 134)	ECD heart and lung recipients (*n* = 65)	p-value
Age, years	45 [34‒54]	43 [33‒53]	41 [33‒49]	47 [35‒56]	50 [39‒54]	**0.019**
Female	232 (51.9%)	102 (57.3%)	33 (47.1%)	68 (50.8%)	29 (44.6%)	0.244
Ethnicity	‒	‒	‒	‒	‒	‒
White	263 (58.8%)	102 (57.3%)	43 (61.4%)	83 (61.9%)	35 (53.9%)	0.667
Black	88 (19.7%)	34 (19.1%)	15 (21.4%)	24 (17.9%)	15 (23.1)	0.822
Hispanic	64 (14.3%)	27 (15.2%)	5 (7.1%)	18 (13.4%)	14 (21.5%)	0.117
Body mass index, kg/m^2^	23.5 [20.8‒27.2]	23.1 [20.1‒26.4]	22.0 [19.5‒25.8]	23.3 [20.9‒27.5]	24.2 [20.9‒28.1]	0.120
Diagnosis	‒	‒	‒	‒	‒	‒
Primary pulmonary hypertension	111 (24.9%)	47 (26.4%)	20 (29.4%)	30 (22.4%)	14 (21.5%)	0.615
Interstitial lung disease	86 (19.3%)	33 (18.5%)	10 (14.7%)	29 (21.6%)	14 (21.5%)	0.598
Cardiomyopathy	58 (13.3%)	25 (14.0%)	7 (10.3%)	15 (11.2%)	11 (16.9%)	0.572
Eisenmenger's syndrome	45 (10.1%)	24 (13.5%)	7 (10.3%)	9 (6.7%)	5 (7.7%)	0.245
Diabetes	65 (14.6%)	27 (15.2%)	3 (4.4%)	21 (15.8%)	14 (21.5%)	**0.040**
Dialysis since listing	22 (5.0%)	9 (5.1%)	3 (4.7%)	6 (4.5%)	4 (6.2%)	0.966
Mechanical ventilation since listing	95 (21.3%)	35 (19.7%)	18 (25.7%)	29 (21.6%)	13 (20.0%)	0.760
ECMO since listing	76 (17.0%)	24 (13.5%)	13 (18.6%)	24 (17.9%)	15 (23.1%)	0.327
IABP since listing	19 (4.3%)	8 (4.5%)	3 (4.3%)	3 (2.2%)	5 (7.6%)	0.356
VAD since listing	17 (3.8%)	5 (1.7%)	4 (5.9%)	8 (6.0%)	2 (3.1%)	0.190
ICU disposition at time of transplant	180 (40.5%)	67 (37.6%)	28 (41.8%)	53 (39.6%)	32 (49.2%)	0.432
Waiting list time, days	75 [22‒209]	96.5 [26‒260]	66 [21‒165]	87.5 [16‒186]	68 [20‒182]	0.363
CMV positive	269 (63.2%)	119 (69.6%)	32 (50.8%)	83 (64.3%)	35 (55.6%)	**0.032**
Previous heart surgery	139 (31.2%)	46 (25.9%)	23 (33.8%)	49 (36.6%)	21 (32.3%)	0.220
Previous lung surgery	14 (3.2%)	3 (1.7%)	3 (4.5%)	5 (3.7%)	3 (4.6%)	0.524
Cigarette use history	133 (29.9%)	53 (29.8%)	14 (20.6%)	45 (33.6%)	21 (32.3%)	0.278
Serum creatinine > 2.0 mg/dL	26 (6.0%)	7 (3.9%)	9 (12.9%)	7 (5.2%)	4 (6.2%)	0.063
Total bilirubin > 2.0 mg/dL	67 (15.0%)	22 (12.4%)	14 (20.0%)	22 (16.4%)	9 (13.9%)	0.454
Cardiac output, L/min	4.0 [3.1‒5.0]	3.8 [3.0‒4.9]	3.8 [3.0‒4.8]	4.1 [3.4‒5.1]	4.4 [3.2‒5.3]	0.230
Mean PA pressure, mmHg	48 [34‒63]	50 [34‒63]	52 [38‒70]	44.5 [34‒65]	45 [27‒60]	0.141
Systolic PA pressure, mmHg	74.5 [52‒96]	79.5 [55‒96]	77 [60‒98]	68 [50‒99]	69 [45‒89]	0.239
Functional deficits	‒	‒	‒	‒	‒	‒
Severe	238 (54.3%)	94 (54.0%)	33 (50.0%)	77 (57.9%)	34 (52.3%)	0.564
Moderate	145 (33.1%)	50 (28.7%)	25 (37.9%)	45 (33.8%)	25 (38.5%)	0.386
Mild to none	55 (12.6%)	30 (17.2%)	8 (12.1%)	11 (8.3%)	6 (9.2%)	0.206
Private payor	255 (57.1%)	105 (59.0%)	41 (58.6%)	74 (55.2%)	35 (53.9%)	0.851
Transplant era						
2005‒2009	128 (28.6%)	66 (37.1%)	29 (41.4%)	26 (19.4%)	7 (10.8%)	**<0.001**
2010‒2014	122 (27.3%)	53 (29.8%)	14 (20.0%)	41 (30.6%)	14 (21.5%)	0.236
2015‒2021	197 (44.1%)	59 (33.2%)	27 (38.6%)	67 (50.0%)	44 (67.7%)	**<0.001**

All values excluding p-values are medians with IQR in brackets or frequencies with prevalence in parentheses. Each p-value is derived from Chi-Squared, Fisher's exact, or Kruskal-Wallis tests.

CMV, Cytomegalovirus; ECD, Extended criteria donor; ECMO, Extracorporeal membrane oxygenation; IABP, Intra-Aortic Balloon Pump; ICU, Intensive Care Unit; LV EF, Left Ventricular Ejection Fraction; PA, Pulmonary Artery; SCD; Standard Criteria Donor; VAD, Ventricular Assist Device.

**Table 3:** Pre-transplant donor demographics of adult heart-lung transplantation recipients from 2005‒2021, stratified by donor organ status.

	All patients	SCD heart and lung recipients	ECD heart only recipients	ECD lung only recipients	ECD heart and lung recipients	p-value
Female	228 (51.0%)	107 (60.1%)	26 (37.1%)	69 (51.5%)	26 (40.0%)	**0.002**
Age, years	31 [21‒43]	29 [20‒43]	24 [19‒33]	35.5 [23‒44]	31 [23‒42]	**0.002**
Body mass index, m/kg^2^	24.4 [21.7‒27.6]	23.6 [21.4‒26.8]	24.2 [20.9‒28.5]	24.7 [22.0‒27.5]	25.6 [23.3‒28.3]	**0.015**
Ethnicity	‒	‒	‒	‒	‒	
White	252 (56.4%)	100 (56.2%)	43 (61.4%)	70 (52.2%)	39 (60.0%)	0.571
Black	68 (15.2%)	23 (12.9%)	15 (21.4%)	22 (16.4%)	8 (12.3%)	0.334
Hispanic	98 (21.9%)	47 (26.4%)	10 (14.3%)	28 (20.9%)	13 (20.0%)	0.195
Hypertension	73 (16.5%)	30 (17.1%)	9 (12.9%)	22 (16.7%)	12 (18.5%)	0.827
Diabetes	20 (4.5%)	10 (5.7%)	1 (1.4%)	9 (6.8%)	0 (0.0%)	0.080
Heavy alcohol use	47 (10.7%)	18 (10.2%)	10 (14.3%)	9 (7.0%)	10 (15.6%)	0.215
On inotropes at procurement	217 (48.8%)	102 (57.3%)	30 (43.5%)	59 (44.0%)	26 (40.6%)	**0.031**
LV EF	60% [55‒65%]	61% [56‒65%]	60% [55‒65%]	60.5% [55‒65%]	60% [55‒65%]	0.570
P/F ratio	450 [369‒509]	461 [390‒517]	471 [393‒520]	437 [344‒488]	423 [334‒497]	**0.001**
Cold ischemic time, hours	3.8 [3.1‒4.4]	3.8 [3.0‒4.4]	3.4 [2.9‒4.3]	3.9 [3.2‒4.4]	3.8 [3.1‒4.2]	0.539
Donor/recipient weight ratio	1.1 [0.9‒1.3]	1.1 [0.9‒1.2]	1.2 [0.8‒1.5]	1.0 [0.9‒1.2]	1.0 [0.8‒1.4]	0.198
Donor/recipient predicted total lung capacity ratio	1.0 [0.9‒1.1]	1.0 [0.9‒1.1]	1.0 [0.9‒1.2]	1.0 [0.9‒1.1]	1.0 [0.9‒1.1]	0.230
HLA mismatches	5 [4‒5]	5 [4‒5]	5 [4‒6]	5 [4‒5]	5 [4‒5]	0.947

All values excluding p-values are medians with IQR in brackets or frequencies with prevalence in parentheses. Each p-value is derived from Chi-Squared, Fisher's exact, or Kruskal-Wallis tests.

ECD, Extended Criteria Donor; HLA, Human Leukocyte Antigen; LV EF, Left Ventricular Ejection Fraction; P/F ratio, Ratio of arterial oxygen partial pressure to fraction of inspired oxygen; SCD, Standard Criteria Donor.

**Table 4:** Pre-transplant extended donor criteria of adult heart-lung transplantations from 2005‒2021, stratified by donor organ status.

	All patients	SCD heart and lung recipients	ECD heart only recipients	ECD lung only recipients	ECD heart and lung recipients	p-value
Age ≥ 55 years	19 (4.3%)	2 (1.1%)	2 (2.9%)	5 (3.7%)	10 (15.4%)	**<0.001**
Cold ischemic time > 6-hours	30 (6.7%)	9 (5.1%)	15 (21.4%)	3 (2.2%)	3 (4.6%)	**<0.001**
Donor/recipient weight ratio 〈0.80 or〉≥ 1.30	156 (34.9%)	36 (20.2%)	50 (71.4%)	30 (22.4%)	40 (61.5%)	**<0.001**
Hepatitis C positivity	9 (2.0%)	1 (0.6%)	1 (1.4%)	0 (0.0%)	7 (10.8%)	**<0.001**
Drug use history	199 (45.0%)	51 (29.0%)	50 (72.5%)	48 (36.4%)	50 (76.9%)	**<0.001**
Serum creatinine > 2.0 mg/dL	55 (12.3%)	6 (3.4%)	21 (30.0%)	6 (4.5%)	22 (33.9%)	**<0.001**
LV EF < 50%	6 (1.3%)	0 (0.0%)	2 (2.9%)	1 (0.8%)	3 (4.6%)	**0.026**
Donation after circulatory death	27 (6.1%)	5 (2.8%)	11 (15.9%)	0 (0.0%)	11 (16.9%)	**<0.001**
Infiltrate on chest radiograph	220 (49.8%)	39 (22.4%)	13 (18.8%)	113 (84.3%)	55 (84.6%)	**<0.001**
P/F ratio < 300	36 (8.1%)	3 (1.7%)	2 (2.9%)	20 (14.9%)	11 (16.9%)	**<0.001**
Purulent secretions on bronchoscopy	72 (16.4%)	9 (5.1%)	3 (4.5%)	43 (32.1%)	17 (27.0%)	**<0.001**
Cigarette use history (> 20 pack-years)	45 (10.3%)	6 (3.4%)	9 (13.0%)	21 (16.3%)	9 (14.1%)	**0.001**

All values excluding p-values are medians with IQR in brackets or frequencies with prevalence in parentheses. Each p-value is derived from chi squared, Fisher's exact, or Kruskal-Wallis tests.

CMV, Cytomegalovirus; ECD, Extended Criteria Donor; LV EF, Left Ventricular Ejection Fraction; P/F ratio, Ratio of arterial oxygen partial pressure to Fraction of inspired oxygen; SCD, Standard Criteria Donor.

For all adults undergoing HLTx from 2005‒2015, one-year survival was 81.6% and five-year survival was 58.5% (Table 5). 120 (27.2%) recipients were diagnosed with a rejection of at least one organ within one year of transplant. 105 patients (25.3%) had a new dialysis requirement post-HLTx and 24 (5.5%) suffered strokes. At 72 h post-HLTx, 103 patients (53.9%) were intubated and 27 (14.1%) were on ECMO. The median hospital length of stay was 28 days (interquartile range 16‒54).

**Table 5:** Morbidity and mortality of adult heart-lung transplantation recipients from 2005‒2021, stratified by donor organ status.

Mortality	All patients	SCD heart and lung recipients	ECD heart only recipients	ECD lung only recipients	ECD heart and lung recipients	p-value
30-day survival function	90.8% [87.7‒93.2]	89.1% [83.5‒92.9]	89.2% [78.7‒94.7]	90.9% [84.5‒94.7]	96.8% [87.9‒99.2]	0.339
One-year survival function	81.6% [77.5‒85.0]	78.3% [71.4‒83.8]	82.7% [70.8‒90.0]	82.8% [75.0‒88.3]	86.7% [74.1‒93.5]	0.394
Five-year survival function	58.5% [52.9‒63.7]	54.6% [46.1‒62.4]	56.1% [41.9‒68.0]	65.8% [55.5‒74.3]	58.1% [38.9‒73.2]	0.428
**Morbidity**						
Rejection within one year	120 (27.2%)	51 (28.7%)	20 (29.9%)	32 (24.1%)	17 (26.6%)	0.777
New dialysis requirement	105 (25.3%)	44 (26.2%)	9 (15.0%)	34 (26.8%)	18 (30.0%)	0.233
Stroke	24 (5.5%)	8 (4.6%)	8 (12.3%)	5 (3.8%)	3 (4.7%)	0.113
PPM placement	8 (1.8%)	0 (0.0%)	2 (3.1%)	3 (2.3%)	3 (4.7%)	**0.019**
Airway dehiscence	6 (1.4%)	4 (2.3%)	1 (1.6%)	0 (0.0%)	1 (1.5%)	0.281
Intubated at 72 h	103 (53.9%)	29 (49.2%)	14 (66.7%)	37 (55.2%)	23 (52.3%)	0.571
On ECMO at 72 h	27 (14.1%)	8 (13.6%)	3 (14.3%)	8 (11.9%)	8 (18.2%)	0.804
Reintubated	119 (27.3%)	49 (28.0%)	18 (28.6%)	32 (24.1%)	20 (30.1%)	0.755
Length of stay, days	28 [16‒54]	25 [16‒51]	33 [14‒63]	28 [18‒52]	36.5 [17‒63.5]	0.562

All values excluding p-values are mortality estimates with 95% confidence intervals in brackets or frequencies with incidence in parentheses, except length of stay which is a median with interquartile range in brackets. Each p-value is derived from Chi-Squared, Fisher's exact, Kruskal-Wallis tests, or log-rank tests.

ECMO, Extracorporeal Membrane Oxygenation; PPM, Permanent Pacemaker.

The four donor organ status groups did not differ in terms of 30-day survival (ranging from 89.1% to 96.8%, *p* = 0.339), one-year survival (which ranged from 78.3% to 86.7%, *p* = 0.394), or five-year survival (which ranged between groups from 54.6% to 65.8%, *p* = 0.428) (Fig. 1). When limiting the analysis to patients who were transplanted from 2011‒2021, the donor organ status groups still did not differ in survival at 30-days (which ranged from 88.5% to 96.1%, *p* = 0.529), 1-year (which ranged from 75.3% to 85.4%, *p* = 0.260), or 5-years (which ranged from 59.1% to 71.1%, *p* = 0.509). These groups also did not differ in terms of early postoperative outcomes including rates of intubation (ranging from 49.2% to 52.3%, *p* = 0.571) and ECMO at 72 h (11.9% to 18.2%, *p* = 0.804), rates of reintubation (24.1% to 30.1%, *p* = 0.755), and median hospital length of stay (25 to 36.5 days, *p* = 0.562). Additionally, the donor organ status groups had similar incidences of complications including rejection within one year of transplant (ranging from 24.1% to 29.9%, *p* = 0.777), new dialysis requirement (ranging from 15.0% to 30.0%, *p* = 0.233), and postoperative stroke (ranging from 3.8% to 12.3%, *p* = 0.113). The groups did however differ in terms of postoperative permanent pacemaker placement with recipients of two ECD organs having the highest rate (4.7%) and recipients of two SCD organs having the lowest (0.0%, *p* = 0.019).

A Cox regression found that donor organ status was not a significant contributor to five-year mortality. It did however identify two predictors of mortality among the extended donor criteria: age ≥ 55 years (HR = 5.52 [2.13‒14.28], *p* < 0.001) and P/F ratio < 300 (HR = 2.38 [1.20‒4.71], *p* = 0.013) (Table 6). No other extended criteria were predictive of five-year mortality on Cox regression.

**Table 6:** Cox regression for five-year mortality of adult heart-lung transplantation recipients from 2005‒2021.

	Hazard ratio	95% confidence interval	p-value
**Donor group**			
ECD heart only	0.87	0.43‒1.79	0.715
ECD lung only	1.08	0.58‒2.01	0.801
ECD heart and lung	0.40	0.14‒1.17	0.093
**Recipient characteristics**			
Age	1.01	0.99‒1.02	0.350
Diabetes	1.28	0.78‒2.07	0.326
CMV positive	0.70	0.48‒1.02	0.060
**Donor characteristics**			
Male sex	0.67	0.47‒0.97	**0.034**
Body mass index, m/kg^2^	1.01	0.98‒1.05	0.459
On inotropes at procurement	1.05	0.73‒1.50	0.809
Transplant year 2005‒2009	1.42	0.91‒2.21	0.119
Transplant year 2015‒2021	0.91	0.58‒1.43	0.690
***Extended donor criteria***			
Age ≥ 55 years	5.52	2.13‒14.28	**<0.001**
Ischemic time > 360 min	0.54	0.23‒1.30	0.171
Donor/recipient weight ratio 〈 0.80 or 〉 1.30	1.13	0.69‒1.83	0.629
Hepatitis C positive	1.78	0.20‒15.79	0.604
Drug use history	1.10	0.70‒1.72	0.682
Serum creatinine > 2.0 mg/dL	1.10	0.59‒2.05	0.769
LV EF < 50%	0.55	0.07‒4.19	0.565
Donation after circulatory death	1.32	0.62‒2.79	0.470
Infiltrates on chest radiograph	0.50	0.30‒0.82	**0.006**
P/F ratio < 300	2.38	1.20‒4.71	**0.013**
Purulent secretions on bronchoscopy	1.00	0.59‒1.68	0.985
Cigarette use history (> 20 pack-years)	1.10	0.62‒1.94	0.740

CMV, Cytomegalovirus; ECD, Extended Criteria Donor; LV EF, Left Ventricular Ejection Fraction; P/F ratio, Ratio of arterial oxygen partial pressure to fraction of inspired oxygen.

## Discussion

Among adults undergoing simultaneous HLTx from 2005 to 2021, the use of an ECD heart and/or lung is not associated with increased mortality at five years, one year, or 30 days. Further, the use of one or more ECD organs is not associated with increased graft rejection, postoperative stroke, or hospital length of stay. The only measure of morbidity positively associated with ECD organ use is postoperative permanent pacemaker placement, which was more likely in recipients of both an ECD heart and an ECD lung. Of the 12 extended donor criteria identified in this study, only two – age ≥ 55 and P/F ratio < 300 – were associated with mortality at 5 years.

The comparable mortality and morbidity between recipients of SCD and ECD organs are not easily explained by recipient characteristics. In fact, recipients of two ECD donor organs were less healthy than other recipients with respect to increased age and diabetes prevalence. The only reported characteristics that may have biased outcomes in favor of recipients of ECD organs were the relative increase in transplantation of ECD organs from 2015‒2021, the higher proportion of SCD organ donors on inotropes at the time of procurement, and the lower rates of cytomegalovirus positivity among recipients of ECD organs. Yet on Cox regression, year of transplant, donor inotrope use, and recipient cytomegalovirus positivity were not significantly associated with five-year mortality. Further, the donor organ status group itself was not associated with five-year survival. It is unclear why recipients of two ECD donor organs had increased pacemaker need, though this may be attributable to these recipients being older [19] and/or have higher rates of donor conduction abnormalities not captured in the UNOS database.

The relative safety of using ECD hearts and ECD lungs is consistent with existing literature showing that the use of ECD hearts or lungs in single-organ transplantation yields comparable outcomes to using SCD organs 1, 2, 3, 4, 5, 6. The present study is unique in demonstrating the safety of ECD organs in the setting of simultaneous HLTx, which is consistent with the literature on single-organ thoracic transplants. Interestingly, the authors found an increase in the relative amount of ECD organs being transplanted in 2015‒2021 relative to previous eras, suggesting that clinical practice is shifting to reflect literature on the tolerability of ECD organs. The present study is also unique in showing the associations between various ECD criteria and heart-lung transplantation outcomes. On Cox regression, the only ECD heart criteria found to significantly contribute to five-year mortality was the age of 55 or greater. The only additional ECD lung criterion found to significantly decrease mortality was P/F ratio < 300. These findings add to the literature suggesting a need to revise ECD heart and lung criteria based on modern outcomes, or otherwise to develop a donor risk score as has been done for single-organ heart transplantation [20,21]. Using a less restrictive set of criteria in the setting of HLTx would likely increase the supply of donor organs given that, at least in the setting of single-organ heart transplantation, donors are often turned down due to ejection fraction less than or equal to 50% or ischemic time greater than four hours [21]. While the present study confirmed that advanced donor age and low P/F ratio are significant risk factors for mortality after HLTx, the authors, nonetheless, advocate that donor selection decisions be made on a holistic basis considering all relevant donor and recipient characteristics.

Limitations of this study are a function of its retrospective design and reliance on the UNOS database. While the UNOS database is invaluable for understanding trends in transplantation, it does not report all pre-transplant variables that are useful for assessing donor organ health nor does it investigate every significant post-transplant outcome. It fails, for example, to report the dose of donor inotrope support at the time of procurement, which is notable given that high-dose inotrope support has been identified as an extended donor criterion. It is thus possible that the present study underestimated the prevalence of ECD organs being used and/or mischaracterized some ECD organ recipients and SCD organ recipients. Additionally, the UNOS database does not report significant post-operative outcomes including primary graft dysfunction, pulmonary function test findings, or echocardiographic measures. Future studies could better characterize the impact of ECD organs on morbidity and mortality by using a prospective design and reporting these additional pre- and post-transplant variables.

## Conclusion

With appropriate patient selection, using one or two ECD organs in HLTx represents a viable strategy for increasing the supply of limited donor hearts and lungs.

## Funding

This research did not receive any specific grant from funding agencies in the public, commercial, or not-for-profit sectors.

## CRediT authorship contribution statement

**Noah Weingarten:** Conceptualization, Data curation, Formal analysis, Methodology, Writing – original draft, Writing – review & editing. **Amit Iyengar:** Conceptualization, Data curation, Formal analysis, Methodology, Supervision, Writing – original draft, Writing – review & editing. **David Alan Herbst:** Writing – original draft, Writing – review & editing. **Mark Helmers:** Writing – original draft, Writing – review & editing. **Danika Meldrum:** Writing – original draft, Writing – review & editing. **Sara Guevara-Plunkett:** Writing – original draft, Writing – review & editing. **Jessica Dominic:** Writing – original draft, Writing – review & editing. **Pavan Atluri:** Conceptualization, Resources, Supervision, Writing – original draft, Writing – review & editing.

## Declaration of Competing Interest

The authors declare no conflicts of interest.
